# Scientific Items

**Published:** 1885-12-20

**Authors:** 


### SCIENTIFIC ITEMS.

**The Pyrophore** —(Popular Science News) At a recent meeting of the Academy of Sciences, at Paris, a plate half filled with water, in which were half a dozen insects about an inch in length, which shone like diamonds, although the room was filled with sunshine, was passed around among the members. These insects had been brought from Mexico, where they are to be found in the forests. Their scientific name is the pyrophore ; and, as none had ever been seen before in Europe, they created quite a sensation. The light resembles that of a glow-worm or a fire-fly, although as much more brilliant and intense as an electric lamp surpasses a wax taper in its power of illumination. When the light begins to fade, it can be made as brilliant as before by shaking the insect, or dipping it in water. It is said the Indians of Mexico use them for a light at night, as a few will suffice to illuminate an entire room. When they are walking at night, they put one on each foot, so that they can be sure of their way, and also that they do not step upon any venomous snake or reptile with which the tropical forests abound. The Mexican ladies buy them of the Indians, and enclose them in a transparent bag, which they wear in their hair or at the neck. The effect is very beautiful, especially when several are worn ; and, as the Indians sell them for a few cents a dozen, they are within the reach of every fair one. They are fed on sugar-cane, and if well taken care of, will live a long time. One placed upon a page will enable it to be read with ease in the darkest night.

**A German** physiologist has recently published some very curious and suggestive observations upon the uniting of different nerves. The experiments were made upon rabbits, cats, dogs, sheep, goats, and pigs ; and the last two were found to bear the operation better than the others. The immediate result, in the case of motor nerves, was the paralysis of the muscle ; but after a time function was restored, at first in a partial way, but later completely. The time necessary to establish normal function varied from three to sixteen months. The first experiments were made upon similar or related nerves, but afterwards they were tried upon those of entirely different functions. The results show that "the central nervous apparatus can influence peripheral organs which do not properly belong to it, provided that it be artificially connected with such peripheral organs." The knowledge is likely to be of some practical value in the future. If certain nerves are injured or destroyed, certain others may be made to do their work, and thus restore a paralyzed organ to its normal activity ; just as telegraphic communication may be re-established by connection with a second wire when one is broken. The experiments indicate that sight and hearing may in like manner be restored, after injury to the nerves on which they directly depend, by bringing other nerves into service by artificial union. These are mere possibilities, but who shall say that they may not become realities at no distant day ?—*Ib.*

**Put Your Light in Front of the Horse.**—Dr. W. B. Ely, in the Philadelphia Reporter, gives his professional brethren the following practical hints :

Few of the accessories to a country doctor's practice are so unsatisfactory as his means of lighting his way when driving at night. The various forms of dash lights are pretty much the same, in that they put the light just where it is not wanted, illuiminating the horse's tail and hips and the buggy-thills with a brilliance quite unnecessary, which intensifies the blackness of the shadow cast by them just where one wishes most to see clearly.

A few nights ago I tried an experiment to overcome this difficulty, which proved such an abundant success that I hasten to give my brethren the benefit of the discovery.

My light is a common tubular lantern, with a reflector, and a spring for attachment to the dash. In place of putting it on the dash, I slipped the spring over the middle of the breast-collar, directly in front of the horse. Every part of the road in front of me was plainly seen, so I could drive with as much confidence as in broad daylight. The conditions necessary for success are a level-headed horse, with fair breadth of chest, and a shoulder-strap attached to the check-hook, to prevent the lantern sagging down between the horse's legs when for any reason the traces slack. It would be well to have a short strap sewed to the inside of the breast-collar, to slip the spring through, so as to prevent any lateral motion.—*Ib.*

**Paper in Tonkin.**—(Gutenberg Journal) The principal material used in the manufacture of paper in Tonkin is the *ke-yioh* or paper tree, which grows in abundance on the mountains in the environs of Sontay. The dried bark of this is brought in bundles upon the backs of oxen or buffaloes from the mountains, where it is gathered for the numerous paper mills, whose principal center is in the vicinity of Hanoi. It is worth about two cents a pound. This bark is macerated and then rubbed up in mortars, so as to reduce it to a fine pulp. This latter is extended with a certain quantity of water in order to form a clear paste, which is sized with an infusion made from the shavings of the *gomao*, a tree which grows in abundance on the Black River mountains.

The paper is manufactured sheet by sheet by women by means of delicate bamboo screens that they alternately dip into the paste and take out therewith a thin sheet of paper, which they deposit upon a board. At the end of the day these sheets are put into a press in order to extract the moisture from them, and are then dried by placing them one by one upon a hot masonry wall. Finally they are put up in packages and trimmed.

Each woman makes a thousand sheets a day. The thickness of the paper depends upon the consistency of the paste. One establishment that was visited by the person who furnished these data was capable of producing 80,000 sheets per day with 80 women and 40 assistants. Paper was being made here worth 65 cents per thousand sheets.

